# Cardioprotective Effects of Sodium Glucose Cotransporter 2 Inhibition in Angiotensin II-Dependent Hypertension Are Mediated by the Local Reduction of Sympathetic Activity and Inflammation

**DOI:** 10.3390/ijms241310710

**Published:** 2023-06-27

**Authors:** Giovanna Castoldi, Raffaella Carletti, Silvia Ippolito, Massimiliano Colzani, Sara Pelucchi, Gianpaolo Zerbini, Gianluca Perseghin, Giovanni Zatti, Cira R. T. di Gioia

**Affiliations:** 1 Dipartimento di Medicina e Chirurgia, Università degli Studi di Milano-Bicocca, 20900 Monza, Italy; m.colzani192@gmail.com (M.C.); sara.pelucchi@unimib.it (S.P.); gianluca.perseghin@unimib.it (G.P.); giovanni.zatti@unimib.it (G.Z.); 2 Dipartimento di Medicina Traslazionale e di Precisione, Sapienza Università di Roma, 00185 Rome, Italy; raffaella.carletti@uniroma1.it; 3 Laboratorio Analisi Chimico Cliniche, Fondazione IRCCS San Gerardo dei Tintori, 20900 Monza, Italy; silvia.ippolito@irccs-sangerardo.it; 4 Unita’ Complicanze del Diabete, IRCCS Istituto Scientifico San Raffaele, 20132 Milano, Italy; zerbini.gianpaolo@hsr.it; 5 Dipartimento di Medicina Interna e Riabilitazione, Policlinico di Monza, 20900 Monza, Italy; 6 Clinica Ortopedica, Fondazione IRCCS San Gerardo dei Tintori, 20900 Monza, Italy; 7 Dipartimento di Scienze Radiologiche, Oncologiche e Anatomopatologiche, Istituto di Anatomia Patologica, Sapienza Università di Roma, 00185 Rome, Italy; cira.digioia@uniroma1.it

**Keywords:** SGLT2-inhibitors, myocardial fibrosis, myocardial hypertrophy, angiotensin II, hypertension, tyrosine hydroxylase, inflammatory infiltrates, rats

## Abstract

The cardioprotective effects of sodium glucose cotrasponter 2 (SGLT2) inhibitors seem to be independent from the effects on glycemic control, through little-known mechanisms. In this study, we investigate whether the cardioprotective effects of empagliflozin, a SGLT2 inhibitor, may be associated with myocardial sympathetic activity and inflammatory cell infiltration in an experimental model of angiotensin II-dependent hypertension. Angiotensin II (Ang II), Ang II plus Empagliflozin, physiological saline, or physiological saline plus empagliflozin were administered to Sprague Dawley rats for two weeks. Blood pressure was measured by plethysmographic method. Myocardial hypertrophy and fibrosis were analysed by histomorphometry, and inflammatory cell infiltration and tyrosine hydroxylase expression, implemented as a marker of sympathetic activity, were evaluated by immunohistochemistry. Ang II increased blood pressure, myocardial hypertrophy, fibrosis, inflammatory infiltrates and tyrosine hydroxylase expression, as compared to the control group. Empagliflozin administration prevented the development of myocardial hypertrophy, fibrosis, inflammatory infiltrates and tyrosine hydroxylase overexpression in Ang II-treated rats, without affecting blood glucose and the Ang II-dependent increase in blood pressure. These data demonstrate that the cardioprotective effects of SGLT2 inhibition in Ang II-dependent hypertension may result from the myocardial reduction of sympathetic activity and inflammation and are independent of the modulation of blood pressure and blood glucose levels.

## 1. Introduction

SGLT2 inhibitors, a widely used class of antidiabetic drugs, induce cardiorenal protection in high-cardiovascular-risk patients [1,2,3,4]. The inhibition of SGLT2, in the renal proximal tubule, promotes glycosuria and natriuresis, leading to a better control of blood glucose level, plasma volume and blood pressure. In diabetic patients with a high cardiovascular risk, the SGLT2 inhibitor empagliflozin reduced the incidence of major cardiovascular events [1] and the progression of kidney diseases [2]. In addition, in both diabetic and non-diabetic patients with heart failure and reduced [5] or preserved ejection fraction [6], empagliflozin reduced cardiovascular death, hospitalization for heart failure and the progression of kidney diseases [5]. Recently, the Empa-kidney trial demonstrated that in patients with chronic kidney diseases of different etiology, empagliflozin treatment reduced the progression of nephropathy and the risk of death for cardiovascular causes [7]. These data strongly suggest that the beneficial effects of SGLT2 inhibition suprass the reduction of blood glucose and could be explained by the pleiotrophic effects of these drugs exerted through several mechanisms, both hemodynamic and non-hemodynamic, which are still not completely understood.

It was therefore hypothesized that the use of SGLT2 inhibitors could be implemented even in the absence of diabetes to counteract organ damage.

Increasing research supports the beneficial effects of SGLT2 inhibition on organ protection in ‘non-diabetic’ diseases. In non-diabetic rats, for example, empagliflozin improved cardiac function after myocardial infarction [8], and in experimental heart failure [9,10]. In Ang II-dependent hypertension [11] and in cyclosporine nephropathy [12], in the absence of diabetes, empagliflozin prevented renal glomerular and tubulointerstitial damage, while in hypertensive N(w)-nitro-L-arginine methyl ester-treated, renin–transgenic (mRen2)27 rats, empagliflozin alone and/or in combination with finerenone, a nonsteroidal mineralocorticoid receptor antagonist, reduced myocardial and renal fibrosis, proteinuria, and allowed to take control of plasma creatinine and blood pressure [13]. Empagliflozin administration improved diastolic dysfunction and myocardial remodeling in Ang II-dependent hypertension [14], preserved the myocardial function in rats with cardiorenal syndrome [15], improved left ventricular function and promoted nephroprotective effects in spontaneously hypertensive rats expressing the human C-reactive protein [16], a non-diabetic model of metabolic syndrome. In these non-diabetic experimental models, the protective effect of SGLT2 inhibition on organ damage appears, al least in part, due to an anti-inflammatory mechanism [11,12,14,15,16]. Modulation of the sympathetic nervous system has been suggested as a potential mechanism of action implicated in the cardioprotective effects of SGLT2 inhibitors [17,18,19,20,21].

In the present study, we investigate, in a model of Ang II-dependent hypertension, whether the beneficial effects of SGTL2 inhibitors on myocardial fibrosis and hypertrophy are mediated by the local modulation of the sympathetic nervous system and inflammation.

## 2. Results

### 2.1. Effects of Ang II and Empagliflozin Administration on Systolic Blood Pressure, Serological Parameters, Heart Weight and Heart/Body Weight Ratio

At the beginning of the study, before starting the different treatments, blood pressure and body weight were similar in the four experimental groups (Control, Control + Empa, Ang II, Ang II + Empa, as described in Supplementary Materials). Angiotensin II administration caused a significant increase in blood pressure in Ang II-treated rats, as compared to the control animals (Table 1).

Empagliflozin administration did not significantly modify blood pressure in control rats and the increase of blood pressure caused by Ang II administration in Ang II-treated rats (Table 1). Body weight was significantly reduced in Ang II + Empa-treated rats, compared to control rats, while Ang II administration alone caused only a slight reduction in body weight in Ang II-treated rats (Table 1). Heart weight was slightly increased in Ang II-treated rats compared to controls, while it did not change in Ang II + Empa treated rats compared to control rats. Heart/body weight ratio was increased both in Ang II-treated rats and Ang II + Empa-treated rats, compared to control and control + Empa-treated rats (Table 1).

Empagliflozin treatment did not significantly modify the non-fasting plasma glucose level both in control and Ang II-treated rats (Table 1). Plasma sodium, potassium, and phosphate did not significantly change among the different groups. Ang II administration did not modify the plasma calcium level, while empagliflozin administration caused a significant decrease in the plasma calcium level, both in control and Ang II-treated rats, compared to control rats (Table 1). Plasma creatinine, cholesterol and triglycerides were similar among the different groups.

### 2.2. Effects of Ang II and Empagliflozin Administration on Myocardial Hypertrophy and Fibrosis

**Figure 1:**
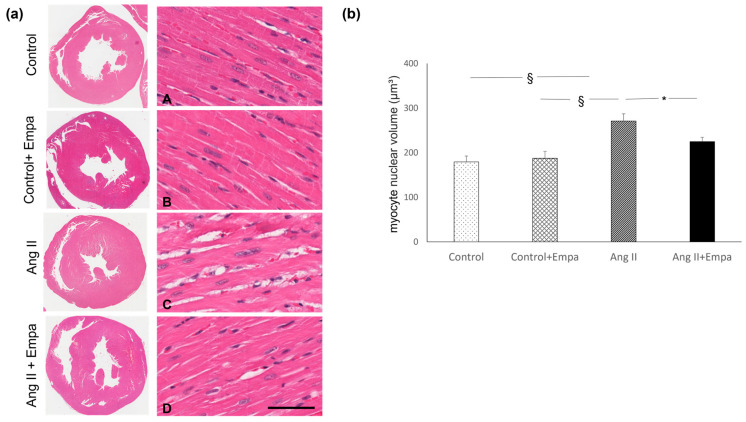
Myocardial hypertrophy. Effect of Empagliflozin administration on myocardial hypertrophy in Ang II-treated rats. (**a**) Representative images of microscopic transverse sections ((**a**) left panel) and related microscopic fields ((**a**) right panel) that show left ventricular hypertrophy with related enlarged cardiomyocyte nuclei. Major and minor nuclear diameters were measured to evaluate the nuclear volume, as described in the Methods. Control (**A**), control + Empa (**B**), Ang II (**C**), Ang II + Empa-treated (**D**) rats (scale bar 100 µm). (**b**) Quantification of myocardial hypertrophy in the different groups of rats. Data are means ± SEM. *: *p* < 0.05. §: *p* <  0.01.

**Figure 2:**
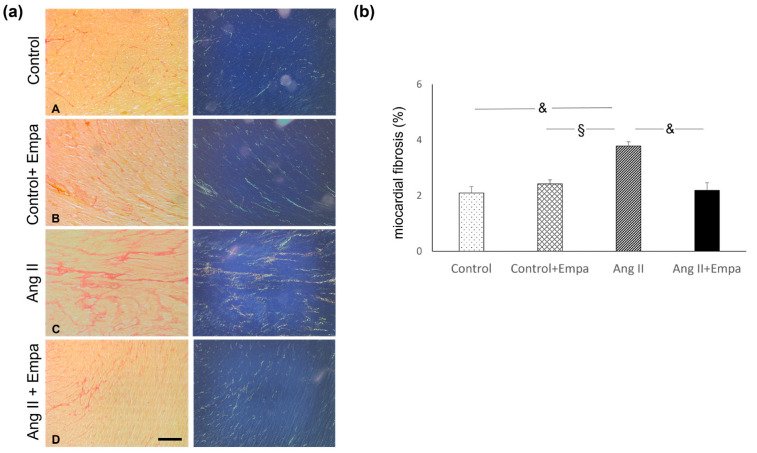
Myocardial interstitial fibrosis. Effects of Empagliflozin administration on myocardial interstitial fibrosis in Ang II-treated rats. (**a**) Representative photomicrographs of interstitial fibrosis (Sirius red stain; ((**a**) left panel)) in control (**A**), control + Empa (**B**), Ang II (**C**), Ang II + Empa-treated (**D**) rats (scale bar 200 µm). Myocardial interstitial fibrosis is caused by an increase in type I collagen fibers, which appear orange/yellow under polarized-light microscopy ((**a**) right panel). (**b**) Quantification of interstitial fibrosis in the different groups of rats. Data are means  ±  SEM. §: *p*  < 0.01. &: *p* <  0.0001.

Ang II administration caused an increase in myocardial hypertrophy and in myocardial interstitial fibrosis, which was prevented by empagliflozin administration (Figure 1 and Figure 2).

Myocardial interstitial fibrosis was characterized by type I collagen fibres, as demonstrated with the analysis under a polarized-light microscope (Figure 2).

### 2.3. Effects of Ang II and Empagliflozin Administration on Myocardial-Inflammatory Cell Infiltration and Tyrosine Hydroxylase Expression

**Figure 3:**
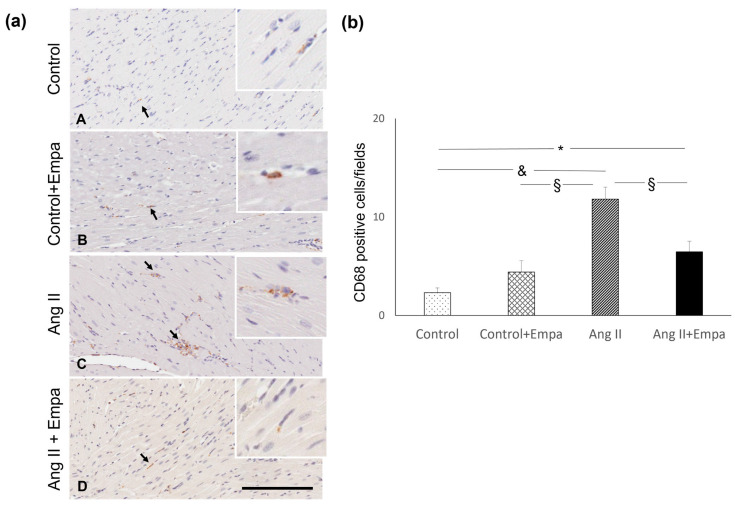
Myocardial inflammatory cell infiltration. Effects of Empagliflozin administration on myocardial inflammatory cell infiltration in Ang II-treated rats. (**a**) Immunohistochemical identification of interstitial monocyte/macrophage infiltration (indicated by the arrows, brown reaction) in control (**A**), control + Empa (**B**), Ang II (**C**), Ang II + Empa-treated (**D**) rats (scale bar 200 µm). (**b**) Quantification of staining of myocardial monocyte/macrophage inflammatory cells in the different groups of rats. Data are means  ±  SEM. * *p* < 0.05. §: *p*  < 0.01. &: *p* < 0.0001.

Ang II administration caused a significant increase in myocardial monocyte/macrophage (CD68 positive cells) infiltration in Ang II-treated rats (Figure 3). Empagliflozin treatment blunted the increase in CD68 positive cells in Ang II-treated rats, compared to rats treated with Ang II alone (Figure 3).

In fact, in Ang II + Empa-treated rats, myocardial CD68 positive cells were significantly higher, compared with control rats (Figure 3).

**Figure 4:**
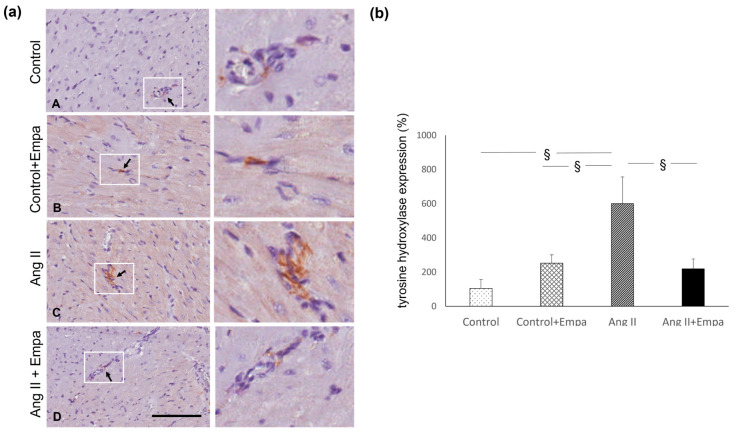
Myocardial tyrosine hydroxylase expression. Effects of Empagliflozin administration on myocardial tyrosine hydroxylase expression in Ang II-treated rats. (**a**) Immunohistochemical identification of tyrosine hydroxylase expression at intraparenchymal nerve fiber level (indicated by the arrows, brown reaction) in control (**A**), control + Empa (**B**), Ang II (**C**), Ang II + Empa-treated (**D**) rats (scale bar 100 µm). The images in the white boxes have been enlarged ((**a**) right panel) (**b**) Quantification of staining of myocardial tyrosine hydroxylase expression in the different groups of rats. Data are means  ±  SEM. §: *p* < 0.01.

Ang II administration caused an increase in myocardial tyrosine hydroxylase expression, which was prevented by empagliflozin treatment (Figure 4).

## 3. Discussion

The results of this study demonstrate that the inhibition of SGLT2 prevented the onset of myocardial hypertrophy and fibrosis in rats with Ang II-dependent hypertension. These effects are mediated by the reduction of inflammatory cells infiltration in the myocardium and by the local reduction of tyrosine hydroxylase expression, an established marker of sympathetic activity.

In an experimental model of Ang II-dependent hypertension, the high blood pressure values, caused by Ang II administration, are the trigger of the development of myocardial hypertrophy and fibrosis. Nonetheless, even if the administration of SGLT2 inhibitor was unable to counteract the onset of hypertension caused by the administration of Ang II, but caused only a slight, but not significant, reduction of blood pressure levels as compared to Ang II-treated group (Table 1), it was nonetheless effective in preventing the development of myocardial damage. This finding strongly supports the hypothesis that the cardioprotective effects of empagliflozin may also occur through non-hemodynamic mechanisms, that is, without the reduction of blood pressure caused by the increase of diuresis and natriuresis, induced by the SGLT2 inhibition. In fact, in our experimental conditions, the reduction of myocardial inflammatory cell infiltration and sympathetic activity are able to counteract the development of myocardial fibrosis and hypertrophy, despite high blood pressure levels. These data are in line with experimental evidences demonstrating that SGLT2 inhibitors might be involved in organ protection through different mechanisms, such as the local reduction of inflammation [11,12,22,23,24,25,26] and a modulation of the sympathetic nervous system [27].

Interestingly, both in diabetic and non-diabetic patients, the beneficial effect of SGLT2 inhibitors has been observed in heart failure, ischemic heart disease, and in chronic kidney disease: disorders which associated themselves to an activation of the sympathetic nervous system [28,29]. Ang II is considered an activator of sympathetic activity and exerts several actions on the sympathetic nervous system promoting sympathetic neurotransmission [30].

Ang II administration stimulates an increase in monocyte/macrophage infiltration, the main cells involved in fibrosis [31], and increases sympathetic activity in the myocardium, which plays a key role in promoting the onset of hypertrophy [32]. These local effects promote, along with the increased blood pressure values, the development of myocardial damage. In our experimental conditions, the administration of SGLT2 inhibitors is able to prevent the development of myocardial alterations, such as hypertrophy and fibrosis, precisely through these two mechanisms, the local reduction of sympathetic activity and inflammation, despite the high blood pressure values caused by Ang II. Interestingly, in a non-diabetic model of hypertension (Ren-2 transgenic rats) empagliflozin administration caused, starting from the fourth week of treatment and up to the end of the protocol at the eighth week, a reduction in blood pressure, reasonably due to the reduction of the sympathetic component of the pressure [33].

Modulation of the TGFβ1/Smad pathway by the SGLT2 inhibitor dapagliflozin has been shown to attenuate myocardial fibrosis and hypertrophy caused by Ang II administration [34]. In line with this evidence, our results also demonstrate that other mechanisms, such as the local modulation of inflammatory infiltrates and sympathetic activity, are able to counteract the development of myocardial damage caused by Ang II infusion. Both in the case of the infusion of Ang II at a dosage of 200 ng/kg/min for 2 weeks, which we used in the present study, and in the case of a dosage of 520 ng/kg/min [34] or 0.4 mg/kg/day [14] for 4 weeks, the cardioprotective effects of the two SGLT2 inhibitors, empagliflozin and dapagliflozin, occur in the presence of high blood pressure values caused by Ang II.

Our data further support the hypothesis that the cardioprotective effects of SGLT2 inhibitors may occur regardless the presence of diabetes. In fact, we studied the effect of empagliflozin in a non-diabetic experimental model, in which myocardial damage cannot be attributed to hyperglycemia. The administration of empagliflozin in our experimental model did not modify blood glucose values, neither in the rats treated with Ang II or those in the control group. These results are in line with the literature data showing that the effects of blood glucose lowering obtained by SGLT2 inhibitors occur only in presence of hyperglycemia [35,36].

This finding could have important clinical implications because it suggests that these drugs can be used independently on their hypoglycemic effect and, especially, in conditions of normo-glycemia, they do not cause hypoglycemia as a side effect, but retain a positive role in organ protection.

In conclusion, our data demonstrate that in Ang II-dependent hypertension, SGLT2 inhibitors have positive effects in counteracting the development of myocardial hypertrophy and fibrosis through mechanisms independent of their effect on glycemia.

## 4. Materials and Methods

### 4.1. Experimental Model of Ang II-Dependent Hypertension

Animal husbandry was in conformity with the Institutional Guidelines in compliance with National laws and policies (D.L.n. 116, Gazzetta Ufficiale della Repubblica Italiana, suppl. 40, 18 February 1992). Experiments were conducted in accordance with the Guide for the Care and Use of Laboratory Animals published by the US National Institutes of Health (NIH Publication No. 85-23, revised 1996). Experiments were performed in conscious male Sprague Dawley rats (10–12 weeks of age), individually housed in cages in a temperature-controlled room with a 12:12 light-dark cycle for the experimental period. One week before the beginning of the protocol, rats were accustomed to experimental procedures. Rats had free access to a standard rat chow and tap water. Body weight (BW, g) was measured once a week. Systolic blood pressure was assessed by the tail-cuff method (average of six recordings, BP Recorder, Ugo Basile Instruments, Gemonio, Italy) at the beginning of any treatment and at the end of the experimental protocol [11,37].

To evaluate the effect of the chronic Ang II administration, osmotic minipumps (Alzet 2002, Palo Alto) were subcutaneously implanted under sodium pentobarbital anesthesia (40 mg/kg/i.p.), in order to administer Ang II (200 ng/kg/min for two weeks, *n* = 8) or physiological saline in the other groups (control group, *n* = 7). Empagliflozin (Empa, 10 mg/kg/day) was administered via being dissolved in drinking water in control + Empa (*n* = 7) and Ang II + Empa-treated rats (*n* = 8). Non-fasting plasma glucose (mg/dL), creatinine (mg/dL), sodium (mEq/L), potassium (mEq/L), calcium (mg/dL), phosphate (mg/dL), cholesterol (mg/dL), and triglycerides (mg/dL) were measured by the colorimetric technique on Cobas Roche (Mannheim, Germany).

At the end of the experimental period, rats were euthanized by an overdose of anesthesia. The hearts were immediately excised, weighted and fixed with 10% formalin, embedded in paraffin and used for light microscopic examination, immunohistochemistry and morphometric analysis to evaluate myocardial hypertrophy and interstitial fibrosis.

### 4.2. Histological Analysis and Morphometric Evaluation of Myocardial Hypertrophy

For all rats, the coronal cardiac sections (3 µm) were deparaffinized, rehydrated, and stained with hematoxylin–eosin (H&E) to evaluate morphological changes in each group studied by light microscopic analysis. The same H&E-stained histological sections were used to assess cardiomyocyte hypertrophy. All slides were captured with an Aperio scanner (Leica Biosystems) and 10 randomly selected images (×40 magnification) were analyzed to evaluate myocardial hypertrophy with a computerized imaging software (ImageJ-win32, FiJi 1.46, IJ1.46r, 2 October 2012, NIH, Bethesda, MD, USA). Major and minor nuclear diameters were measured to evaluate the nuclear volume (V) using the formula for a prolate ellipsoid: V = pAB2/6, where A is the major diameter and B the minor diameter [38]. Fifty nuclei from each rat were measured [39].

### 4.3. Histological and Morphometric Analysis of Myocardial Fibrosis and Collagen Analysis by Polarized Light Microscopy

Consecutive histological cardiac sections (4 µm) were stained with Sirius Red, a collagen-specific stain, and used for morphometric analysis of myocardial fibrosis, as previously described [39]. Briefly, all slides were captured with an Aperio scanner (Leica Biosystems, Buccinasco, taly) and 20 randomly selected images (×20 magnification) were analyzed to evaluate interstitial collagen volume fraction with computerized imaging software (Image J-win32, NIH, Bethesda, MD, USA), and expressed as the ratio between red-stained interstitial area and the total area of the heart section. The same Sirius Red-stained sections were analyzed using a light microscope under polarized light to evaluate the different types of collagen as previously described [40]. For each sample, 20 randomly selected microscopic fields were analyzed with a polarized-light microscope (Leitz Camera, Leiz, Oberkochen, Germany) using a ×20 magnification. Images were captured by a computerized digital camera (Olympus Camedia 5050, Olympus, Center Valley, PA, USA) using SPOT (Diagnostic Instruments, Sterling Heights, MI, USA), and analyzed for different types of collagen. Image analysis was performed by two pathologists blinded to the experimental source of the samples [40].

### 4.4. Immunohistochemical Evaluation of Monocyte/Macrophage Infiltration and Myocardial Tyrosine Hydroxylase Expression

The count of monocytes/macrophages infiltration was performed on formalin fixed and paraffin embedded transmural myocardial sections (3 μm), using a monoclonal mouse anti-rat monocytes/macrophage (CD68, clone ED1, MAB 1435, Chemicon, Temecula, CA, USA). The histological sections were deparaffinized and rehydrated, treated by boiling in citrate buffer (0.01 mol/L, pH 6) in a microwave (750 W), and incubated over night at 4 °C with a primary antibody (1:300). The reaction product was amplified by Ultra Tek HRP Staining System (Scy TeK Laboratories, Logan, UT, USA) and visualized with 3,3′-diaminobenzidine (DAB) (Dako, Glostrup, Denmark). Negative control was obtained by omitting the primary antibody. Sections were viewed using a Leica microscope (Leitz Camera), and all slides with immunostained myocardial section for each sample were captured with an Aperio scanner (Leica Biosystems). Twenty randomly selected images/section at ×20 magnification were analyzed. Two independent pathologists blinded to the treatment counted the CD68 positive cells and obtained the average. The macrophages were expressed as a mean value of positive cells/fields [39].

The tyrosine hydroxylase expression was evaluated on formalin-fixed and paraffin-embedded myocardial sections (3 µm) using an anti-rabbit Anti-Tyrosine Hydroxylase antibody—Neuronal Marker (ab112 Abcam, Cambridge, UK). The sections were deparaffinized and rehydrated, treated with Proteinase K (20 µg/mL; Qiagen, Hilden, Germany) for 10 min at 37 °C, and successively incubated with the primary antibody (1:1000, for an hour). The reaction product was amplified by the Ultra Tek HRP Staining System (Scy TeK Laboratories, Logan, UT, USA) and visualized with 3,3′-diaminobenzidine (DAB) (Dako, Glostrup, Denmark). Negative control was obtained by omitting the primary antibody. Sections were viewed using a Leica microscope (Leitz Camera), and all slides with immunostained myocardial section for each sample were captured with an Aperio scanner (Leica Biosystems). Ten randomly selected images/section at ×40 magnification were analysed by two independent pathologists blinded to the treatment. The tyrosine hydroxylase immunostaining at nerve fibers was expressed as a percentage (immunostaining area/total histological area) [39].

### 4.5. Statistical Analysis

Data are shown as means ± SEM (standard error of the mean). Differences among the groups of rats (control, control + Empa, Ang II, Ang II + Empa) for systolic blood pressure, body weight, heart weight, heart/body weight, blood glucose, sodium, potassium, calcium, phosphate, creatinine, cholesterol, triglycerides, myocardial fibrosis and hypertrophy, monocyte/macrophage infiltrates, and tyrosine hydroxylase expression were assessed using ANOVA, followed by Fisher’s protected least-significant test for post-hoc comparisons. Differences between means were considered significant at *p* < 0.05.

## Figures and Tables

**Table 1:** Systolic blood pressure (SBP, mmHg), body weight (BW, g), heart weight (g), heart/body weight ratio (mg/g), non-fasting plasma glucose (mg/dL), sodium (mEq/L), potassium (mEq/L), calcium (mg/dL), phosphate (mg/dL), creatinine (mg/dL), total cholesterol (mg/dL), triglycerides (mg/dL) in Control (*n* = 7), Control + Empa (*n* = 7), Ang II (*n* = 8), Ang + Empa (*n* = 8)-treated rats at the end of the two week experimental period.

Parameters	Control	Control + Empa	Ang II	Ang II + Empa
SBP (mmHg)	142.9 ± 3.0	145.4 ± 2.0	202.5 ± 2.3 † ‡	196.1 ± 2.4 † ‡
BW (g)	388.0 ± 15.4	383.2 ± 8.4	361.8 ± 8.3	350.0 ± 6.4 * ζ
Heart Weight (g)	1.26 ± 0.05	1.12 ± 0.02	1.37 ± 0.05 δ	1.28 ± 0.04 ζ
Heart/Body Weight (mg/g)	3.26 ± 0.15	2.94 ± 0.05	3.78 ± 0.18 * δ	3.67 ± 0.14 δ
Plasma				
Glucose (mg/dL)	161.0 ± 11.0	143.5 ± 8.1	166.0 ± 10.2	168.5 ± 7.2
Sodium (mEq/L)	140.9 ± 1.02	139.0 ± 1.47	139.9 ± 0.76	139.5 ± 0.78
Potassium (mEq/L)	3.76 ± 0.31	4.66 ± 0.76	3.88 ± 0.35	3.73 ± 0.26
Calcium (mg/dL)	8.94 ± 0.22	7.97 ± 0.41 *	8.19 ± 0.21	7.77 ± 0.15 §
Phosphate (mg/dL)	6.76 ± 1.24	7.11 ± 0.89	6.67 ± 0.51	6.79 ± 0.56
Creatinine (mg/dL)	0.22 ± 0.01	0.24 ± 0.03	0.25 ± 0.02	0.29 ± 0.03
Cholesterol (mg/dL)	59.8 ± 4.67	59.2 ± 3.07	56.1 ± 3.20	56.4 ± 2.68
Triglycerides (mg/dL)	78.0 ± 25.8	124.0 ± 15.6	101.8 ± 12.3	86.1 ± 13.0

Data are means  ±  SEM. * = *p* < 0.05 vs. Control; § = *p* < 0.01 vs. Control; ‡ = *p* < 0.0001 vs. Control; ζ = *p* < 0.05 vs. Control + Empa; δ = *p* < 0.01 vs. Control + Empa; † = *p* < 0.0001 vs. Control + Empa.

## Data Availability

The data presented in this study are available on request from the corresponding author.

## Supplementary Materials

The following supporting information can be downloaded at: https://www.mdpi.com/article/10.3390/ijms241310710/s1.
