# Supplementary material for: Cardioprotective Effects of Sodium Glucose Cotransporter 2 Inhibition in Angiotensin II-Dependent Hypertension Are Mediated by the Local Reduction of Sympathetic Activity and Inflammation
Source: Int J Mol Sci. 2023 Jun 27;24(13):10710. doi: 10.3390/ijms241310710 (PMC10341774; doi:10.3390/ijms241310710)
Supplement: Supplementary file 1 [file ijms-24-10710-s001.zip › ijms-2443065-supplementary.pdf]

Supplementary Materials

Systolic Blood Pressure and Body Weight at the beginning of the experimental protocol.

Before starting the experimental protocol, rats were divided in four groups (Control, Control+Empa, Ang II, Ang II+Empa). Body weight (g) and Systolic Blood Pressure (mmHg) were measured as described in the Methods. No differences were present among the different groups.

**Parameters**	**Control**	**Control + Empa**	**Ang II**	**Ang II + Empa**
SBP (mmHg)	143.7 ± 4.0	149.1 ± 1.0	141.5 ± 4.3	140.2 ± 2.7
BW (g)	253.6 ± 14.0	270.7 ± 5.0	261.2 ± 6.3	253.1 ± 5.8
